# Effect of Bergmeister papilla on disc parameters in spectral domain optical coherence tomography

**DOI:** 10.1038/s41433-023-02818-z

**Published:** 2023-11-18

**Authors:** Yeoun Sook Chun, Nam Ju Moon, Ungsoo Samuel Kim, Joon Hyung Yeo, Jae Hoon Jeong

**Affiliations:** 1 Department of Ophthalmology, Chung-Ang University Hospital, Seoul, Korea; 2 Department of Ophthalmology, College of Medicine Chung-Ang University, Seoul, Korea; 3 Department of Ophthalmology, Chung-Ang University Gwangmyeong Hospital, Gwangmyeong City, Gyeonggido, Korea

**Keywords:** Eye manifestations, Eye diseases

## Abstract

**Objectives:**

To investigate the morphological characteristics of Bergmeister papilla (BMP), a persistent hyaloid remnant tissue, and its effects on the measurements and repeatability of spectral-domain optical coherence tomography (OCT).

**Subjects/Methods:**

The subjects of this prospective cross-sectional study including 83 patients with BMP and 76 unaffected individuals, all had open-angle structures. Images, including a 5-line raster and three consecutive optic disc cube scans centred on the optic disc, were acquired using the Cirrus high-definition OCT. BMP’s structural characteristics were classified based on the raster scan images, and repeatability of acquiring optic nerve head and retinal nerve fibre layer parameters acquisition was analysed by calculating the test-retest standard deviation (*S*w), coefficient of variance (CV), and intraclass correlation coefficient.

**Results:**

BMPs (*n* = 83) were categorised into lifting edge (LE) type (63.9%, *n* = 53), which partially covers the edge of the optic nerve head, and covering disc (CD) type (36.1%, *n* = 30), which completely covers the cupping area like a cap. The average cup-to-disc ratio (0.58 ± 0.21), vertical cup-to-disc ratio (0.55 ± 0.21), and cup volume (0.22 ± 0.22) of the CD type were lower than those of the LE type (0.66 ± 0.13, 0.64 ± 0.13, and 0.4 ± 0.27, respectively; all *P* < 0.05). Tolerability indices for repeatability of cup volume (*S*w = 0.40 and CV = 0.36) and inferonasal (4 o’clock) retinal nerve fibre layer (*S*w = 0.27 and CV = 0.25) in LE-type BMPs exceeded the cut-off value (0.22) and demonstrated stronger correlation with BMP location than that of the controls.

**Conclusion:**

Caution should be exercised when interpreting OCT findings in eyes with BMP, as BMP can introduce a pitfall in OCT imaging.

## Introduction

Bergmeister papilla (BMP) is a remnant of the hyaloid artery fibrous sheath, which is usually asymptomatic and incidentally observed during fundus examinations. It is characterised by avascular prepapillary veils and epipapillary membranes. Various types of BMP can be identified during fundus examination [1, 2]. However, the morphological characteristics of BMP, as revealed using optical coherence tomography (OCT), and its effects on OCT remain unclear.

Incomplete atrophy of the hyaloid artery during embryonic and post-embryonic development leads to persistent hyaloid remnants; their reported prevalence varies across studies. Paediatric studies using traditional ophthalmoscopy following pupil dilation have reported a prevalence of 3% in mature infants and 95% in premature infants [3]. Conversely, a study using OCT reported a prevalence of 67.8% in full-term myopic children with normal growth and development [4]. Among adults, the prevalence was 0.03 − 0.8% when using fundus photography [5, 6], whereas it was 50% when OCT was used [7]. In a clinical examination of 100 eyes of 50 young adults (aged 20−30 years), slit lamp and ophthalmoscopy could not identify BMP, whereas 84% of spectral OCT scans detected its presence [8]. This difference may be attributed to OCT’s enhanced visualisation capacity at the vitreoretinal interface and posterior vitreous cortex [7]. Persistence of BMP is not an uncommon ophthalmic condition, considering its prevalence rate of 32.14% in an autopsy study [9].

Currently, OCT is invaluable for assessing structural changes in eyes with pathological conditions, because it provides quantitative and non-invasive in vivo assessments of macular and optic nerve head (ONH) parameters [10–12]. Additionally, the advent of spectral-domain (SD) OCT has improved image resolution, image speed, and sensitivity [13–15]; however, many potential artefacts are still present in OCT imaging, which are related to ocular pathologic features and non-ocular errors, including technical and software errors [16]. Epiretinal membrane, posterior vitreous detachment, retinoschisis, high myopia, and media opacities have been reported as representative ocular pathological features associated with segmentation errors in OCT imaging [16, 17].

Furthermore, BMP can pose difficulties in interpreting clinical data obtained from OCT because it closely adheres to the surface of the ONH. This study aimed to reveal the BMP’s characteristics using SD-OCT and evaluate its influence on parameters concerning disc and peripapillary retinal nerve fibre layer (RNFL), particularly focusing on the repeatability of OCT measurements.

## Materials and Methods

This was a prospective cross-sectional observational study. The Institutional Review Board of the Konyang University Hospital, Daejeon, Korea approved the study protocol (IRB No. KYUH 2021-07-013-001). All procedures were performed in accordance with the tenets of the World Medical Association’s Declaration of Helsinki, and written informed consent was obtained from all participants.

### Study participants

Participants were selected from patients referred to an outpatient clinic for further ophthalmologic evaluation at the Health Screening Center, Konyang University Hospital, Daejeon, Korea between July 2021 and June 2022. A comprehensive initial ophthalmic examination was conducted, including assessment of best-corrected visual acuity; intraocular pressure (IOP) using a Goldmann applanation tonometer; noncycloplegic autorefraction; and examination of the anterior segment using silt-lamp, gonioscopy, fundoscopy (using a 90 D lens), and non-mydriatic fundus photography (VX-10, Kowa Optimed, Tokyo, Japan). Exclusion criteria encompassed patients with best-corrected visual acuity <20/40 according to the Snellen chart, IOP > 21 mmHg, cylinder correction <–3.0 D or >+3.0 D (as astigmatism greater than 3.25 D may have a clinically significant effect on OCT interpretation) [18], closed-angle gonioscopy findings, diagnosis of ophthalmologic diseases affecting visual functions other than mild dysfunctional tear syndrome (Level 1 according to the Delphi Panel Report) [19], and history of ocular surgery other than uncomplicated cataract extraction or pterygium excisional surgery within 6 months before this study.

Patients with open-angle structures were categorized based on funduscopy and fundus images into those with BMP and those with no specific structural changes on the ONH. When both eyes of a patient had BMP or were unaffected (control subjects), only one eye was randomly chosen for inclusion. To locate BMP, we divided the ONH into superior, inferior, nasal, and temporal quadrants, following the RNFL quadrant classification convention using the Cirrus high-definition OCT (HD-OCT; Carl Zeiss Meditec, Dublin, CA, USA).

### OCT measurements

Two high-definition image scans (HD 5-line raster protocol) in the horizontal and vertical directions and three consecutive peripapillary RNFL scans centred on the ONH (optic disc cube 200×200 protocol) were acquired without pupil dilation by the same operator on the same day. The ONH and peripapillary RNFL thickness parameters were automatically measured using the respective analysis algorithms.

The optic disc cube 200×200 program generated ONH images from a 6×6×2 mm dataset using 200×200 axial scans. The system extracted a B-scan from the data in a 3.46-mm diameter circle, and the algorithm automatically detected the circle around the ONH [20]. After identifying the anterior and posterior boundaries of the RNFL, the system calculated RNFL thickness at each point along the circle.

High-definition images were evaluated for BMP’s structural characteristics, while rim area, disc area, average cup-to-disc ratio (C/D), vertical C/D, and cup volume of the ONH were analysed for repeatability. Additionally, we evaluated the average, superior, inferior, temporal, and nasal quadrant thicknesses with 12-clock-hour thickness in peripapillary RNFL analysis. For convenience, locations were described based on the right eye, with 12, 3, 6, and 9 o’clock representing the superior, nasal, inferior, and temporal quadrants, respectively. Participants with scans showing signal strength below six or artefacts resulting from eye movements or blinking were excluded from the study. Segmentation errors within the retinal pigment epithelium layer of optic nerve B-scans and RNFL of peripapillary RNFL B-scans were excluded.

### Statistical analysis

To evaluate the effect of BMP on consecutive OCT scans (repeatability), three types of statistical analyses were performed: mean within-subject standard deviation (*S*w), calculated as the square root of the average of each variance, derived by one-way ANOVA; coefficient of variation (CoV), expressed as the percentage of the standard deviation divided by the mean; and intraclass correlation coefficient (ICC), which was centred and scaled using a pooled mean and standard deviation. Tolerance index was used to compare the BMP and control groups, as described by Bergin et al [21]. Briefly, this was computed as the log of the ratio between the repeatability of a pathological cohort and that of a healthy population. Tolerance index *T* was calculated using the following equation:

$$T{r}_{i}={\log }_{n}\left(\frac{{r}_{{P}_{i}}}{{r}_{{H}_{i}}}\right)$$

where *r* is repeatability, *i* is the OCT parameter, and *P* and *H* correspond to the pathological cohort and healthy population, respectively. Therefore, a *T* value of 0 represents a perfect alignment between the respective limits of repeatability, and a larger divergence from 0 indicates greater divergence between limits. Bergin et al. established *T* cutoff values based on sample size because the statistical significance of divergence depends on sample size. For instance, when comparing between groups of 30 and 50 eyes, the tolerance index cut-off is 0.28; however, when a group of 30 eyes is compared to a larger sample size of 75 eyes, the cutoff drops to 0.26. Similarly, when comparing a group of 75 eyes to 50 eyes, the tolerance index cut-off is 0.22, which reduces to 0.20 against a larger sample size of 75 eyes.

Differences in demographic and clinical characteristics between patients with BMP and unaffected individuals were assessed using the Student’s *t*-test, one-way ANOVA and chi-square test. Post-hoc analyses employed Tukey’s and Scheffé’s tests. Statistical significance was set at 5%. Data were recorded and analysed using SPSS for Windows (version 20.0; SPSS Inc., Chicago, IL, USA).

## Results

**Fig. 1:**
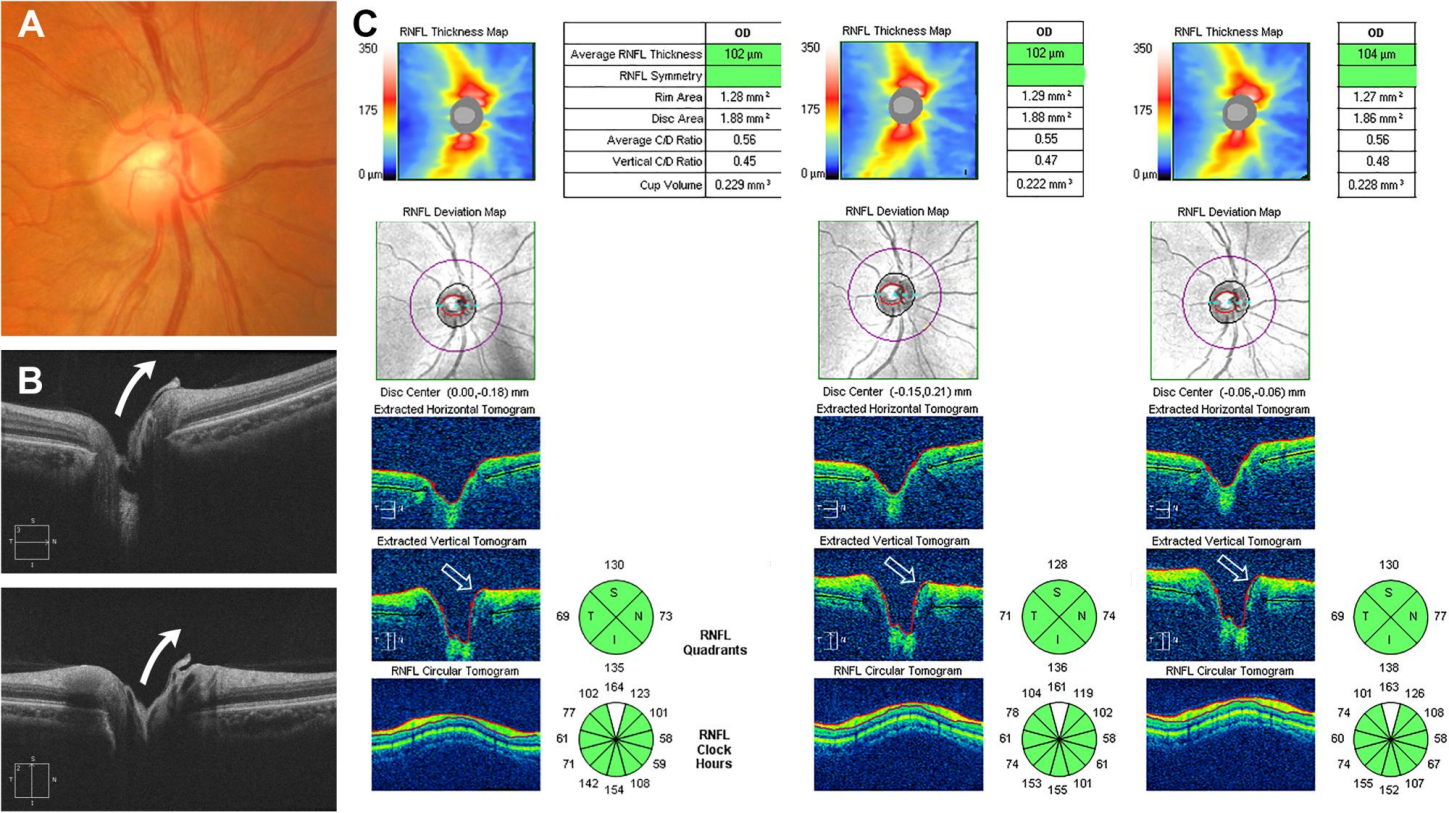
Representative case of the lifting edge type of Bergmeister papilla. **A** Fundus photograph of the right eye with a Bergmeister papilla (BMP) superonasal to the optic disc. **B** Horizontal (upper image) and vertical (lower image) high-definition optical coherence tomography images showing a persistent hyaloid remnant tissue that ascends along the neuroretinal rim of the optic disc (arrows). **C** Three consecutive optic disc cube scans centred on the optic disc showing that the automatic segmentation of the internal limiting membrane (red line) is raised in the area where the BMP is located (empty arrows).

**Fig. 2:**
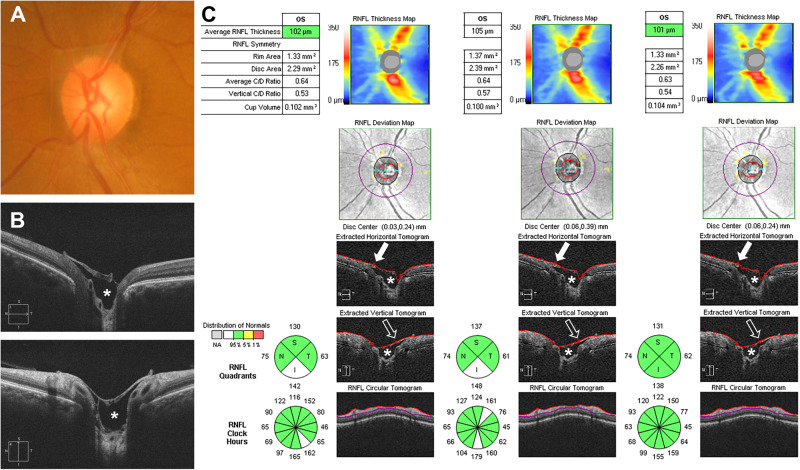
Representative case of the covering disc type of Bergmeister papilla. **A** Fundus photograph of the left eye with a Bergmeister papilla (BMP). **B** Horizontal (upper image) and vertical (lower image) high-definition optical coherence tomography images showing a persistent hyaloid remnant tissue covering the entire cupping area of the optic disc like a cap. **C** Identification of the neuroretinal rim in the nasal (arrows) and superior (empty arrows) quadrants is affected by the BMP; therefore, automatic segmentation of the internal limiting membrane (red line) appears different from the normal condition in three consecutive optic disc cube scans. Empty spaces (asterisks) are identified between the BMP and anterior lamina tissue when using the raster and the optic disc cube protocols.

BMP was confirmed through OCT in all participants exhibiting raised glial tissue on the ONH’s surface on fundus examination and photography. Based on the OCT image analysis of BMP, we classified the cases into two categories. The first type was the lifting edge (LE) type, with BMP not entirely covering the ONH but ascending along the neuroretinal rim of the ONH, and partially covering its corners in the raster scan (Fig. 1). The second type was the covering disc (CD) type, with BMP entirely covering the cupping area of the ONH like a cap (Fig. 2). Cases where an empty space was observed between the BMP and anterior lamina tissue in the horizontal and vertical tomograms of the raster protocol or within the optic disc cube protocol were classified as CD type. Automatic segmentation of the internal limiting membrane (ILM) (red line) near the BMP for both types exhibited differences from the actual structure during the optic disc cube scans.

Overall, 159 eyes from 159 participants (83 eyes with BMP and 76 normal control eyes) were included in this study. Demographic and quantitative measurements of ocular characteristics from the BMP and unaffected control groups are summarized in Table 1. Morphological classification of BMP revealed a higher prevalence of the LE type (*n* = 53) than the CD type (*n* = 30), and no significant differences in age, sex, best-corrected visual acuity, spherical equivalent, or IOP were observed among the three groups, including the normal control group. Topographical analysis was not performed for the CD-type BMPs, as they spanned across more than three quadrants. Conversely, the LE-type BMPs were more frequently located in the nasal (35 eyes, 66.0%) than in the inferonasal (8 eyes, 15.1%), inferior (5 eyes, 9.4%), superior (2 eyes, 3.8%), superonasal (2 eyes, 3.8%), and temporal (1 eye, 1.9%) quadrants of the ONH.

**Table 1:** Demographic and ocular characteristics from the BMP and unaffected control groups.

	Lifting edge type (*n* = 53)	Covering disc type (*n* = 30)	Control (*n* = 76)	*P*^*a*^
Age, mean (SD), years	52.9 (16.1)	52.4 (11.8)	49.8 (16.4)	0.513
Male Sex (%)	28 (52.8)	16 (53.3)	33 (43.4)	0.481
BCVA, mean (SD), logMAR	0.02 (0.05)	0.06 (0.25)	0.02 (0.05)	0.223
SE, mean (SD), Dioptre	−1.21 (2.51)	−1.86 (2.23)	−1.45 (2.22)	0.476
IOP, mean (SD), mmHg	15.72 (2.62)	16.22 (3.19)	15.77 (2.81)	0.715
Topographical distribution of BMP (%)				
Superior Superonasal Nasal Inferonasal Inferior Temporal	2 (3.8%) 2 (3.8%) 35 (66.0%) 8 (15.1%) 5 (9.4%) 1 (1.9%)	N/A	N/A	N/A

^a^ ANOVA is performed for analysing items excluding sex, and the chi-square test is performed for analysing sex.

*BCVA* best-corrected visual acuity, *MAR* minimum angle of resolution, *SE* spherical equivalent, *IOP* intraocular pressure, *BMP* Bergmeister papilla, *SD* standard deviation.

The OCT parameters are listed in Table 2. No statistically significant differences were detected in OCT parameters between the BMP and the control groups. However, subgroup analysis revealed significant differences in several ONH parameters between the two BMP types. The average and vertical C/Ds were significantly lower in the CD group than in the other groups (*P* < 0.05). The cup volume was largest in the LE, control, and CD groups, with a significant difference only between the CD and LE groups (*P* < 0.05). No significant differences in RNFL parameters, including the four quadrants and the 12 clock-hour domains, were observed among the three groups.

**Table 2:** OCT parameters from the BMP and unaffected control groups.

	BMP (*n* = 83)			Control (*n* = 76)	*P*^*a*^	*P*^*b*^
	Lifting edge type (LE) (*n* = 53)	Covering disc type (CD) (*n* = 30)	Both types			
Signal strength	6.55	6.51	6.53	6.53	0.994	0.944
Optic nerve head parameters						
Rim area, mean, mm^2^	1.07	1.13	1.09	1.09	0.419	0.707
Disc area, mean, mm^2^	2.07	1.91	2.01	2.06	0.276	0.177
Average C/D	0.66	0.58	0.63	0.65	0.064	0.028 (LE vs CD: 0.041)^c^ (CD vs Control: 0.040)^c^
Vertical C/D	0.64	0.55	0.61	0.62	0.148	0.019 (LE vs CD: 0.019)^c^ (CD vs Control: 0.048)^c^
Cup volume, mean, mm^3^	0.396	0.223	0.333	0.337	0.273	0.015 (LE vs CD: 0.011)^c^
Retinal nerve fibre layer parameters, mean, μm						
Average thickness	87.61	85.30	86.78	88.30	0.455	0.556
Superior quadrant	107.94	108.54	108.16	111.31	0.351	0.644
Nasal quadrant	65.08	62.46	64.13	62.64	0.330	0.313
Inferior quadrant	110.01	103.54	107.67	112.00	0.217	0.205
Temporal quadrant	67.30	66.88	67.14	67.70	0.784	0.955
12 o’clock hour	107.35	113.93	109.73	113.07	0.474	0.486
1 o’clock hour	97.24	98.77	97.79	97.25	0.888	0.955
2 o’clock hour	76.05	72.82	74.88	73.23	0.486	0.508
3 o’clock hour	57.47	57.36	57.43	55.58	0.191	0.433
4 o’clock hour	61.64	56.94	59.94	59.07	0.608	0.141
5 o’clock hour	86.97	82.70	85.43	88.11	0.610	0.411
6 o’clock hour	118.33	109.98	115.31	115.96	0.889	0.451
7 o’clock hour	124.52	117.96	122.15	131.91	0.087	0.085
8 o’clock hour	71.32	69.76	70.76	68.23	0.345	0.593
9 o’clock hour	53.62	54.60	53.97	54.64	0.532	0.827
10 o’clock hour	78.13	76.31	77.47	79.14	0.995	0.751
11 o’clock hour	123.59	112.78	119.68	119.34	0.165	0.116

^a^Comparison between both types of BMP and the unaffected control group by the Student’s *t*-test.

^b^Comparison between each type of BMP and the unaffected control group by one-way ANOVA.

^c^Post-hoc analysis values using Tukey’s method.

*OCT* optical coherence tomography, *BMP* Bergmeister papilla, *C/D* cup-to-disc ratio.

The repeatability of the OCT parameters is presented in Table 3. Regarding repeatability, the cup volume exhibited variability in BMP eyes than in control eyes. The cup volume measurements were as follows: control eyes (*n* = 76), *S*w = 0.022 and CoV=6.59%; both BMP types (*n* = 83), *S*w = 0.043 and CoV=14.44%. *Tr* values of Sw and CoV, comparing control and BMP eyes, were Log_n_(0.043/0.022) = 0.28 and Log_n_(14.44/6.59) = 0.34, respectively. These values exceeded the 0.2 *Tr* cut-off for differentiating between control and BMP eyes (both types) based on sample size [21], indicating poor repeatability of cup volume measurements in BMP eyes. Among the RNFL parameters, only the 4 o’clock hour thickness demonstrated worse repeatability in BMP eyes than in control eyes; *Tr* values of Sw and CoV were 0.21 (=Log_n_[5.77/3.52]) and 0.21 (=Log_n_[9.63/5.97]), respectively.

**Table 3:** Repeatability of the OCT parameters from the BMP and unaffected control groups.

	BMP (*n* = 83)									Control (*n* = 76)		
	Lifting edge type (*n* = 53)			Covering disc type (*n* = 30)			Both types					
	Sw	CV	ICC	Sw	CV	ICC	Sw	CV	ICC	Sw	CV	ICC
Optic nerve head parameters												
Rim area	0.07	6.07	0.936	0.05	4.31	0.995	0.06	5.50	0.964	0.07	6.35	0.960
Disc area	0.09	4.20	0.979	0.06	3.06	0.993	0.08	3.86	0.985	0.09	4.50	0.985
Average C/D	0.02	2.79	0.994	0.02	3.16	0.997	0.02	2.92	0.996	0.02	2.56	0.990
Vertical C/D	0.03	4.27	0.972	0.02	4.04	0.996	0.02	4.21	0.986	0.02	3.75	0.983
Cup volume	0.051^a, b^	15.12^a^	0.988	0.022	9.78	0.997	0.043^a^	14.44^a^	0.991	0.022	6.59	0.997
Retinal nerve fibre layer parameters												
Average thickness	2.44	2.78	0.989	2.23	2.62	0.995	2.37	2.73	0.992	2.47	2.80	0.975
Superior quadrant	4.64	4.30	0.985	3.92	3.61	0.995	4.40	4.06	0.990	3.77	3.38	0.980
Nasal quadrant	4.46	6.82	0.956	3.67	5.93	0.954	4.01	6.25	0.956	2.93	4.65	0.967
Inferior quadrant	3.77	3.43	0.991	4.01	3.87	0.993	3.86	3.59	0.992	3.84	3.43	0.986
Temporal quadrant	2.13	3.16	0.989	1.97	2.95	0.997	2.07	3.09	0.994	2.14	3.16	0.986
12 o’clock hour	7.37	6.86	0.980	7.41	6.50	0.986	9.11	8.30	0.983	5.97	5.28	0.982
1 o’clock hour	5.49	5.64	0.983	6.17	6.25	0.989	5.74	5.87	0.986	6.50	6.68	0.966
2 o’clock hour	6.15	8.08	0.949	6.93	9.51	0.945	6.44	8.60	0.947	4.30	5.87	0.967
3 o’clock hour	4.00	6.96	0.954	4.39	7.66	0.907	4.15	7.22	0.941	3.63	6.52	0.928
4 o’clock hour	6.56^a^	10.64^a^	0.920	4.01	7.04	0.920	5.77^a^	9.63^a^	0.922	3.52	5.97	0.952
5 o’clock hour	5.37	6.18	0.979	4.16	5.03	0.987	4.97	5.82	0.982	5.35	6.07	0.963
6 o’clock hour	6.14	5.19	0.987	6.55	5.95	0.988	6.29	5.45	0.987	5.41	6.07	0.985
7 o’clock hour	5.99	4.81	0.985	6.77	5.74	0.990	6.28	5.14	0.987	6.22	4.71	0.984
8 o’clock hour	4.29	6.29	0.975	2.93	4.20	0.996	3.85	5.60	0.987	4.51	6.32	0.960
9 o’clock hour	2.27	4.16	0.975	2.16	3.96	0.994	2.23	4.09	0.987	1.76	3.29	0.986
10 o’clock hour	3.50	4.42	0.983	3.61	4.73	0.991	3.54	4.53	0.987	3.02	3.86	0.984
11 o’clock hour	7.17	6.01	0.975	6.20	5.50	0.986	6.84	5.84	0.980	5.15	4.17	0.978

^a^The tolerance index value is greater than the cut-off in the BMP group than in the control group.

^b^The tolerance index value is greater than the cut-off in the lifting edge type than in the covering disc type.

*BMP* Bergmeister papilla, *Sw* within-subject standard deviation, *CV* coefficient of variation, *ICC* intra-correlation coefficient, *C/D* cup-to-disc ratio.

In the subgroup analysis, the LE type exhibited a pattern similar to that of the overall BMP eyes. The cup volume measurements were as follows: LE-type BMPs (*n* = 53), *S*w = 0.051 and CoV=15.12%; and CD-type BMPs (*n* = 30), *S*w = 0.022 and CoV=9.78%. *Tr* values for Sw between the control and BMP eyes were Log_n_(0.051/0.022) = 0.40 in the LE type and Log_n_(0.022/0.022) = 0 in the CD type. *Tr* values for CoV between the control and BMP eyes were Log_n_(15.12/6.59) = 0.36 in the LE type and Log_n_(9.78/6.59) = 0.17 in the CD type. The respective *Tr* cut-off values calculated according to sample size, were 0.22 and 0.26 between the control and LE groups and between the control and CD groups, respectively [21]. *Tr* values for the CD type were less than the cut-off value, whereas those for the LE type were greater than the cut-off value, indicating worse repeatability of cup volume measurements in LE-type BMPs than those of CD-type BMPs. Among the RNFL parameters, only the 4 o’clock hour thickness exhibited worse repeatability in the LE-type BMPs than in the control eyes; *Tr* values of Sw and CoV were 0.27 (=Log_n_[6.56/3.52]) and 0.25 (=Log_n_[10.64/5.97]), respectively. However, all *Tr* values obtained from the comparison of other RNFL parameters between the control and BMP eyes were less than the cut-off value.

Only the cup volume demonstrated worse repeatability in the LE-type BMPs compared with CD-type BMPs: *Tr* values of *S*w and CoV were 0.40 (=Log_n_[0.05/0.02]) and 0.19 (=Log_n_[15.12/9.78]), respectively. Considering the sample sizes were 30 (CD type) and 53 (LE type), the corresponding *Tr* cut-off value was 0.28 [21]. A significant difference in repeatability based on *S*w was observed between the CD- and LE-type BMPs, whereas *Tr* fell below the cut-off value based on the CoV.

According to the ICC guidelines reported by Koo and Li [22], all the index values across various groups were >0.90, indicating excellent agreement, and all *Tr* values of ICC fell below the cut-off level in inter-group comparison. Similar patterns were observed in most groups. Among ONH parameters in all groups, except for the CD type, the ICC values of the rim area were the lowest at 0.936, 0.964, and 0.960 in the LE type, both BMP types, and control groups, respectively. Among the average RNFL quadrant thickness parameters, the ICC values of the nasal quadrant were the lowest at 0.956, 0.954, 0.956, and 0.967 in the LE type, CD type, both BMP types, and control groups, respectively. Among the 12-clock-hour thickness RNFL parameters, the ICC values at 3 and 4 o’clock were the lowest at 0.920, 0.907, 0.922, and 0.928 in the LE type, CD type, both BMP types, and control groups, respectively.

## Discussion

In this study, we investigated BMP’s characteristics and its effects on SD-OCT images. BMP can be divided into the LE type, partially lining the neuroretinal rim of the optic nerve, and the CD type, entirely covering the cup of the ONH, creating an empty space. The LE type was more prevalent than the CD type and was most commonly located in the nasal quadrant. Our findings confirmed that BMP could affect autosegmentation of the ILM, although its overall effect on OCT was marginal. The CD type affected the C/D and cup volume measurements, whereas the LE type affected the repeatability of cup volume and nasal RNFL parameters. To the best of our knowledge, no reports discussing the influence of BMP on SD-OCT scans are indexed in PubMed. Furthermore, these findings may have implications for vitreopapillary traction syndrome [23], a disorder of the vitreous-papillary interface characterised by the fibrocellular vitreal membrane pulling at adherent sites on the optic disc, or proliferative diabetic retinopathy exhibiting various forms of optic disc neovascularization on OCT [24].

Previous studies have classified BMP based on histological characteristics [25] or regression features on OCT images [4]. These embryological classifications may be useful in paediatric patients. A case report described a patient aged > 50 years with asymptomatic BMP exhibiting optic nerve elevation with traction causing macular schisis on OCT [26], suggesting that complete absorption of the hyaloid artery is unlikely to occur in adults. In this study, we analysed the effect of asymptomatic BMP incidentally detected on OCT scans. Since invasive biopsies are not feasible in asymptomatic individuals, we divided the cases into CD and LE types based on the presence of empty space between the BMP and ONH on the raster scans.

Among the ONH parameters, the disc area revealed no significant variation between the groups, whereas the mean and vertical C/Ds were significantly smaller in the CD group (Table 2), indicating a smaller cup boundary. A special algorithm was applied for autosegmentation of each OCT device. The algorithm used in Cirrus OCT determined the optic disc edge based on the termination point of the Bruch’s membrane and calculated the rim width around the optic disc’s circumference by measuring the minimum cross section between the Bruch’s membrane opening and the ILM. As shown in Fig. 2, BMP impacts autosegmentation of the ILM in the CD type, possibly causing the quantified rim area to appear larger than its actual size, although no statistically significant difference was observed. Therefore, the cup boundary possibly appeared smaller in this group than in other groups, and the reduced volume might have been associated with ILM segmentation errors. Although the C/D may lack clinical significance as a single indicator, the error factor should be considered, because C/D is commonly analysed in glaucoma diagnosis to identify pathological ONH; this trend may hold relevance in identifying progression.

Although ophthalmoscopy and photography have been used successfully in clinical settings and trials, they remain subjective with low-to-moderate interobserver agreement and limited sensitivity to detect subtle changes [27]. OCT offers a more objective and quantitative evaluation of the optic disc, and Cirrus OCT measurements of ONH parameters and RNFL thickness demonstrate no difference in distinguishing between normal and glaucomatous eyes [28]. However, many ocular pathological features can cause segmentation errors in OCT imaging [16, 17], and the present findings suggested the BMP might be one of them. Considering the absence of an ideal optic disc evaluation method, it has been suggested that measurements obtained by clinicians and those derived from OCT are still not interchangeable in clinical practice.

Understanding the factors affecting repeatability is crucial because the degree of measurement noise often changes with disease stage or pathology type [29]. When comparing the average values of ONH parameters, the CD group exhibited differences in cup-related factors compared to other groups; however, after applying the concept of ‘*T’* to assess repeatability, only the cup volume of the LE group exhibited lower repeatability. In high-definition image scans, the LE type was attached to a part of the optic disc, and this attachment affected the ILM autosegmentation due to a slight elevation at the end of the vitreous cavity. Although a quantitative analysis was not performed, the structural characteristics of the vitreous end of the BMP may be related to the lower repeatability in the LE type.

Regarding the RNFL parameters, only the 4 o’clock thickness of the LE type exhibited significantly low repeatability compared with that of the control eyes, potentially due to the orientation of BMP relative to the ONH. Our study revealed that >90% of LE types displayed attachment to the nasal and inferior quadrants with minimal distribution in the superior and temporal quadrants, even if superonasal (3.8%) locations were included in the superior quadrants. These findings aligns with the results observed by Roth et al [9]. in autopsied eyes (74.5% and 24.2% in the nasal and inferonasal quadrants, respectively) and Lin et al [4]. who studied myopic children using swept-source OCT (72.2% and 22.39% in the nasal and inferior quadrants, respectively). This distribution pattern is associated with the low repeatability of the 4 o’clock thickness, corresponding to the nasal and inferior quadrants, where LE-type BMP is predominantly located.

Although this study was conducted on healthy eyes, the effect of BMP on the monitoring of glaucoma progression remains unclear. Previous studies have indicated excellent short-term reproducibility for circumpapillary RNFL measurements using Cirrus OCT. Leung et al [30]. reported intra- and inter-visit ICCs of 0.975 and 0.963, respectively, in healthy individuals, and Mwanza et al [31]. reported intra- and inter-visit ICCs of 0.972 and 0.986, respectively, in patients with glaucoma. Consistent with these results, our findings revealed ICC values of 0.975 and 0.992 in the unaffected control and BMP groups, respectively, suggesting that the coexistence of BMP and glaucoma does not significantly affect the repeatability of OCT measurement. However, both glaucoma [31] and BMP eye evaluations demonstrated the lowest reproducibility in the nasal quadrant, indicating that errors may have affected the relevant parameters.

High reproducibility is crucial when selecting ideal parameters. When BMP and glaucoma coexist, selecting the best parameter for monitoring glaucoma progression can be challenging. In this study, one of each ONH and RNFL parameters in the LE type affected repeated measurements based on the concept of *T*. Although circumpapillary RNFL thickness is the most widely used parameter, its relative values may vary across different disease stages due to the floor effect[32]. Moreover, determining the superiority of one parameter over another is challenging due to an incomplete understanding of the temporal relationship between changes in the ONH and RNFL [33]. Therefore, complementary combinations of ONH and RNFL parameters increases sensitivity in monitoring glaucoma progression in the presence of BMP.

This study had some limitations. First, the findings were obtained from highly specific population (Korean). Additional data acquisition and verification across different ethnic groups are required for a more generalizable interpretation. Second, the classification and attachment location analysis of BMP were constrained due to the use of a horizontal and vertical HD 5-line raster protocol. Although conducting multiple raster scans in different directions might have provided a solution, practical constraints limited additional OCT scans. Therefore, developing a method for a more detailed morphological analysis of BMP, such as 3D reconstruction, is required. Third, OCT measurements in this study were performed by a single observer, potentially introducing observer bias [34]. In general, blinding becomes less important in reducing observer bias when outcomes are less subjective [35], because objective OCT data offer minimal opportunity for bias.

In conclusion, this study classified asymptomatic BMPs into two types based on their morphological characteristics using OCT and demonstrated their effects on OCT parameters. Although establishing definite clinical significance remains challenging, our findings confirmed that BMP affects ILM autosegmentation. BMP presents a potential pitfall warranting cautious interpretation of OCT data, particularly when assessing cup volume and nasal RNFL thickness for diagnosing and discriminating the progression of optic nerve disease.

## Summary

### What was known before

- Bergmeister papilla is a remnant of the hyaloid artery fibrous sheath.
- Bergmeister papilla is usually asymptomatic and is observed incidentally during fundus examinations.
- Analysis of the morphological characteristics of Bergmeister papilla using OCT or the effect on OCT has not been fully clarified.

### What this study adds

- Bergmeister papilla was categorized into lifting edge and covering disc type.
- Some of the optic nerve head parameters in OCT showed significant differences between the covering disc and lifting edge types and normal control eyes.
- Repeatability of the lifting edge types was influenced by cup volume and nasal retinal nerve fibre layer parameters.

## Data Availability

The datasets generated during and/or analysed during the current study are available from the corresponding author on reasonable request.
